# Comparison of surgical outcomes of C1-2 fusion surgery between O-arm-assisted operation and C-arm assisted operation in children with atlantoaxial rotatory fixation

**DOI:** 10.3389/fped.2023.1059844

**Published:** 2023-02-20

**Authors:** Xin Zhou, Yue Benny Yang, Yichen Meng, Tao Lin, Xuhui Zhou, Ce Wang

**Affiliations:** ^1^ Department of Orthopedics, Second Affiliated Hospital of Naval Medical University, Shanghai, China; ^2^ Department of Endocrine and Metabolic Diseases, Shanghai Institute of Endocrine and Metabolic Diseases, Ruijin Hospital, Shanghai Jiao Tong University School of Medicine, Shanghai, China; ^3^ Shanghai National Clinical Research Center for Metabolic Diseases, Key Laboratory for Endocrine and Metabolic Diseases of the National Health Commission of the PR China, Shanghai Key Laboratory for Endocrine Tumor, State Key Laboratory of Medical Genomics, Ruijin Hospital, Shanghai Jiao Tong University School of Medicine, Shanghai, China

**Keywords:** o-arm, atlantoaxial rotatory fixation, navigation, children, pedicle screws

## Abstract

**Objective:**

Placement of the pedicle screw is technically challenging during C1-2 fusion surgery in children and different intraoperative image-guided systems have been developed to reduce the risk of screw malposition. The purpose of the present study was to compare surgical outcomes between C-arm fluoroscopy and O-arm navigated pedicle screw placement in the treatment of atlantoaxial rotatory fixation in children.

**Methods:**

We retrospectively evaluated charts of all consecutive children with atlantoaxial rotatory fixation who underwent C-arm fluoroscopy or O-arm navigated pedicle screw placement from April 2014 to December 2020. Outcomes including operative time, estimated blood loss (EBL), accuracy of screw placement (Neo's classification) and completed fusion time were evaluated.

**Results:**

A total of 340 screws were placed in 85 patients. The accuracy of screw placement of the O-arm group was 97.4%, which was significantly higher than that of the C-arm group (91.8%). Both groups had satisfied bony fusion (100%). Statistical significance (230.0 ± 34.6 ml for the C-arm group and 150.6 ± 47.3 ml for the O-arm group, *p* < 0.05) was observed with respect to the median blood loss. There were no statistically significant difference (122.0 ± 16.5 min for the C-arm group and 110.0 ± 14.4 min for the O-arm group, *p *= 0.604) with respect to the median operative time.

**Conclusion:**

O-arm-assisted navigation allowed more accurate screw placement and less intraoperative blood loss. Both groups had satisfied bony fusion. O-arm navigation did not prolong the operative time despite the time required for setting and scanning.

## Introduction

The atlantoaxial joint is the most mobile joint of the spine because of the horizontally oriented facet joints, the lax ligaments and the robust synovium that allow hypermobility (1). But these anatomic features also predispose this complex to a disorder called atlantoaxial rotatory fixation (AARF). In children, the larger head-to-body mass ratio and the undeveloped bone structures exacerbate the incidence of AARF (2). Children with AARF typically present with torticollis, facial asymmetry with oral malocclusion, and limited neck motion (3). The primary treatment of AARF in children is conservative, while surgery is recommended when conservative treatment fails (4).

With the development of modern techniques and instruments, the atlantoaxial posterior screw-rod technique has become the gold standard of the posterior fixation technique for AARF (5). This has been widely recognized by spine surgeons because of its advantages in long-term stability, high fusion rates, and excellent correction (6, 7). However, the placement of the pedicle screw is technically challenging for pediatric patients due to the small size of the pedicle, high variation, and high risk of neurovascular injury (8). Different intraoperative image-guided systems have been developed to reduce the risk of screw malposition and achieve safe fixation in cervical spine surgery (9). Traditionally, intraoperative C-arm fluoroscopy has been used during surgery. However, the pitfalls of C-arm fluoroscopy include single-plane imaging, insufficiently clear imaging, and high occupational radiation exposure (10). Therefore, just as surgical techniques have advanced over the years, imaging modalities have also.

The O-arm navigation system (Medtronic® Sofamor Danek, Minneapolis, MN, USA), a three-dimensional intraoperative imaging device, has gained worldwide popularity since its first introduction in 2008 (11, 12). However, there are no reports on the feasibility and efficacy of O-arm navigation-guided pedicle screw placement in the treatment of children with atlantoaxial rotatory fixation. In this study, we compared O-arm navigation with C-arm fluoroscopic guidance in surgical outcomes of children with atlantoaxial rotatory fixation.

## Methods and materials

We retrospectively reviewed the medical records of consecutive patients who underwent C1–C2 fusion between April 2014 and December 2020. The study was reviewed and approved by the Institutional Review Board of Second Affiliated Hospital of Naval Medical University. All patients were subjected to instrumentation utilizing the Resnick and Benzel's procedure (6). for C1–C2 fixation performed under fluoroscopic guidance of the C-arm or navigation of the O-arm.

### Preoperative preparation

Each patient received preoperative cervical traction for an average of 3 days. Surgery was recommended for those who were resistant to closed reduction (13, 14). Radiographic examinations, including plain and dynamic flexion-extension x-rays, computed tomographic (CT) scans and magnetic resonance imaging (MRI) were routinely performed. Three-dimensional printed models based on computed tomography angiography (CTA) were built to evaluate the patency of the vertebral artery.

### Operative approach

The patients were placed in a prone position with a radiolucent plaster bed to secure the head under general anesthesia. Cranial traction (with a maximum of one sixth body weight) was performed to reduce the dislocation as much as possible. A posterior midline incision was made to expose the posterior region of the C1-2 complex. The surgical technique for the fluoroscopic guided group was previously described (6). Regarding the navigation-guided group, the navigation reference frame was mounted on the C2 spinous process and lateral and anterior–posterior (AP) views were taken to determine scan position. An O-arm scan was performed to obtain 3-dimensional (3D) images, which were then transferred to the StealthStation navigation system (Medtronic, Inc., Minneapolis, Minnesota, United States) for automatic registration. The scanned images were directly transferred with the navigation devices and auto-regulation was performed. The registered probe was used to confirm the accuracy of the navigation, and the entry point and orientation of the trajectory were selected based on the axial, coronal and sagittal images of the StealthStation. The initial pilot holes were drilled with a high-speed drill, followed by the navigated pedicle drill to maintain course. A ball-tip probe was then used to confirm the integrity of the trajectory and to ensure that no cortical penetration into the spinal canal had occurred. The optimal screw length was then measured using the navigation system and the screws were placed under image guidance. Then, bilateral rods were placed in the screw heads, and the C2 screws were first tightened. Using the height difference between the C1 and C2 screws, the rotated C1 was lifted by the lever system and appropriately retracted or compressed to further reduce rotation. A second intraoperative O-arm scan was obtained to evaluate the position of the screws. Additional scanning was performed to adjust the screw position in some complicated cases. The posterior arch of the C1 and C2 lamina were decorticated with a high-speed drill and an autogenous bone graft taken from the iliac crest was placed on the bone fusion bed. Neuromonitoring of the sensory and motor evoked potentials was used throughout the surgical procedure.

### Outcome measures

Variables including operative time, estimated blood loss (EBL), screw placement accuracy, intra- and postoperative complications, length of stay, and complete fusion time were recorded.

A CT scan at postoperative day 1 was taken to confirm the position of the screws. Screw accuracy was assessed using Neo's classification (15):. Grade 0, without deviation, the screw was contained in the pedicle; Grade 1, deviation less than 2 mm (i.e., less than half of the screw diameter); Grade 2, deviation more than 2 mm and less than 4 mm; Grade 3, deviation more than 4 mm (i.e., complete deviation). The screw accuracy rate was defined as the number of screws classified as Grade 0 and 1/the total number of screws.

Solid bone fusion was assessed on a 12-month CT-scan, and was defined as the presence of continuous bridging trabecular bone between the dorsal elements of C1 and C2.

### Statistical analysis

Statistical analyzes were performed with SPSS version 21.0 (SPSS, INC., Chicago, IL, USA) and the *p* value < 0.05 was considered as a statistical significance. Normalized data were compared using the unpaired *t* test, with data expressed as means ± standard deviation. Fisher's exact test and the Chi-square test were used to analyze categorical data.

## Results

Eighty-five patients who underwent C1-2 fusion were included in the present study: 46 in the C-arm and 39 in the O-arm groups. Mean age at surgery was 9.67 years (range, 6–15). The mean follow-up was 12 months (range 13–16) (Table 1).

**Table 1:** Comparison of the patients' demographic characteristics.

Parameters	C-arm fluoroscopy group (*n* = 46)	O-arm navigation group (*n* = 39)	*p*-value
Age	9.46 ± 3.22	9.92 ± 3.66	0.533
Gender (male: female)	20: 26	22: 17	0.235
BMI	16.65 ± 2.51	16.79 ± 2.13	0.780

A total of 340 screws were inserted. On postoperative CT-scan, the screw accuracy rate of the O-arm group was 97.4%, which was significantly higher than that of the C-arm group (91.8%, *p *= 0.025), details shown in Table 2.

**Table 2:** Accuracy of pedicle screws in both groups.

Group		Number of screws	Grade 0	Grade 1	Grade 2	Grade 3
C-arm fluoroscopic group	C1	92	69	13	9	1
	C2	92	74	13	5	0
	Total	184	143 (77.7%)	26 (14.1%)	14 (7.6%)	1 (0.5%)
O-arm navigation group	C1	78	70	5	2	1
	C2	78	73	4	0	1
	Total	156	143 (91.2%)	9 (5.8%)	2 (1.3%)	2 (1.3%)

EBL showed a statistical difference between C-arm and O-arm groups with 230.0 and 150.6 ml respectively, *p* < 0.05. No statistically significant difference (122.0 ± 16.5 min vs. 110.0 ± 14.4 min, *p *= 0.604) was observed regarding the median operative time between the two groups, details shown in Table 3.

**Table 3:** Comparison of the operative data.

Parameters	C-arm fluoroscopy group	O-arm navigation group	*p*-value
EBL (ml)	230.0 ± 34.6	150.6 ± 47.3	0.032
Operative time (minutes)	172.0 ± 36.5	170.0 ± 14.6	0.708
Length of stay (days)	3.5 ± 1.3	3.9 ± 2.1	0.520

No serious complications such as vascular complications, spinal cord injury, or cerebrospinal leak occurred and, the postoperative course was uneventful for all patients. The average length of stay was 3.5 days (range 3–6). Both groups had satisfied bony fusion (100%) at 12 months of follow-up.

## Discussion

In this study, we showed that the accuracy of pedicle screw placement in C1-2 fusion surgeries using the O-arm guidance was higher than those using C-arm. Ling et al. reported that 98% of their screws were placed in ideal positions in 21 patients treated with a Harm's construct or occipital cervical fusion using the O-arm (16). We achieved similar results in the present study. With the use of intraoperative navigation, the trajectory of each screw could be easily visualized to ensure that nerve roots, spinal cord or vertebral arteries were not damaged.

Another benefit of O-arm navigation was that it could reduce intraoperative blood loss. Hitti et al. (17) reported a significant reduction in EBL, by more than 50% using O-arm navigation in C1-2 posterior cervical fixation, which was consistent with our results. This finding was likely explained by the minimal disturbance of the cervical venous plexus. Typically, surgeries performed with navigation required a limited exposure due to image guidance of the hardware placement and, therefore, less need for extensive dissection for direct visualization.

More importantly, as the pedicle size in children is small, repeated adjustment during screw placement could lead to screw loosening, which would seriously affect the pullout strength. Navigation with the O-arm provided the precision to insert all C1 and C2 screws in a single path, which technically also translated into greater bony purchase and biomechanical stability. Additionally, because of interference from the mandible and teeth that overlap, conventional fluoroscopic techniques cannot clearly assess the degree of reduction due to the relationship between the odontoid and the lateral mass. The advent of O-arm navigation has overcome this shortcoming by providing axial CT images during navigation, which clearly shows the position of the odontoid in relation to the lateral mass, as well as the foramen transversarium and spinal canal in relation to the pedicle screws. Another point, when navigated with fluoroscopy, interpretation of the screw trajectory requires constant switching between the lateral and antero–posterior views. O-arm navigation based on simultaneous guidance by axial, sagittal, and coronal views allowed the positions of the screws to be updated in real time as they advanced into the vertebrae. Therefore, we could check the trajectory and position of the screw to reduce the possibility of reoperation due to mispositioned screws.

Prolonged operative time was another potential problem with O-arm navigation due to the time required to set up and scan. Hitti et al. reported that posterior fixation of C1–C2 with O-arm navigation (198.4 ± 13.56 min) takes longer than with the C arm fluoroscopy (156.9 ± 10.03 min) (17). However, they also reported that with increased experience and familiarity with navigation equipment, there was a decrease in the duration of the procedure and eventually, similar operative times were spent between the surgery with O-arm navigation and that with the C arm fluoroscopy. In this study, there were also no statistically significant differences in median operative time between the groups. The reason for this was likely that our surgeons minimized the exposure of bony landmarks for screw placement and insert the screws with confidence using the O-arm navigation. Fixation assisted by C-arm fluoroscopy required meticulous dissection of bony landmarks such as the lateral portion of the posterior arch of C1 and the isthmus of C2. O-arm-assisted fixation could cut these steps short and reduce operative time.

This study was limited by its retrospective design and the non-randomization of patients. Since the introduction of the O-arm in our institution, only a few C-arm surgeries have been performed. Furthermore, surgery for pediatric C1-2 fusion was relatively rare and required a steep learning curve, which was a limitation for large-scale studies. Further studies will be required to reach a more definitive conclusion.

## Conclusion

O-arm-assisted navigation allowed more accurate screw placement and less intraoperative blood loss in pediatric C1-2 fixation, compared to a conventional fluoroscopic guided device. Using O-arm navigation did not prolong the operative time despite the time required for setting and scanning.

## Data Availability

The raw data supporting the conclusions of this article will be made available by the authors, without undue reservation.

## References

[1] Beier AD Vachhrajani S Bayerl SH Aguilar CY Lamberti-Pasculli M Drake JM. Rotatory subluxation: experience from the hospital for sick children. J Neurosurg Pediatr. (2012) 9:144–8. 10.3171/2011.11.PEDS11147 22295918

[2] Attia W Orief T Almusrea K Alfawareh M Soualmi L Orz Y. Role of the O-arm and computer-assisted navigation of safe screw fixation in children with traumatic rotatory atlantoaxial subluxation. Asian Spine J. (2012) 6:266–73. 10.4184/asj.2012.6.4.266 23275810 PMC3530701

[3] Neal KM Mohamed AS. Atlantoaxial rotatory subluxation in children. J Am Acad Orthop Surg. (2015) 23:382–92. 10.5435/JAAOS-D-14-00115 26001430

[4] Yang SY Boniello AJ Poorman CE Chang AL Wang S Passias PG. A review of the diagnosis and treatment of atlantoaxial dislocations. Global Spine J. (2014) 4:197–210. 10.1055/s-0034-1376371 25083363 PMC4111952

[5] Desai R Stevenson CB Crawford AH Durrani AA Mangano FT. C-1 lateral mass screw fixation in children with atlantoaxial instability: case series and technical report. J Spinal Disord Tech. (2010) 23:474–9. 10.1097/BSD.0b013e3181bf9f24 20124915

[6] Resnick DK Benzel EC. C1–C2 pedicle screw fixation with rigid cantilever beam construct: case report and technical note. Neurosurgery. (2002) 50:426–8. 10.1097/00006123-200202000-00039 11844283

[7] Hojo Y Ito M Suda K Oda I Yoshimoto H Abumi K. A multicenter study on accuracy and complications of freehand placement of cervical pedicle screws under lateral fluoroscopy in different pathological conditions: CT-based evaluation of more than 1,000 screws. Eur Spine J. (2014) 23:2166–74. 10.1007/s00586-014-3470-0 25047653

[8] Gluf WM Brockmeyer DL. Atlantoaxial transarticular screw fixation: a review of surgical indications, fusion rate, complications, and lessons learned in 67 pediatric patients. J Neurosurg Spine. (2005) 2:164–9. 10.3171/spi.2005.2.2.0164 15739528

[9] Park P Foley KT Cowan JA Marca FL. Minimally invasive pedicle screw fixation utilizing O-arm fluoroscopy with computer-assisted navigation: feasibility, technique, and preliminary results. Surg Neurol Int. (2010) 1:44. 10.4103/2152-7806.68705 20975974 PMC2958329

[10] Ojodu I Ogunsemoyin A Hopp S Pohlemann T Ige O Akinola O. C-arm fluoroscopy in orthopaedic surgical practice. Eur J Orthop Surg Traumatol. (2018) 28:1563–8. 10.1007/s00590-018-2234-7 29796825

[11] Shin HK Jeon SR Roh SW Park JH. Benefits and pitfalls of O-arm navigation in cervical pedicle screw. World Neurosurg. (2022) 159:e460–5. 10.1016/j.wneu.2021.12.077 34958990

[12] Shin MH Hur JW Ryu KS Park CK. Prospective comparison study between the fluoroscopy-guided and navigation coupled with O-arm-guided pedicle screw placement in the thoracic and lumbosacral spines. J Spinal Disord Tech. (2015) 28:E347–51. 10.1097/BSD.0b013e31829047a7 23563342

[13] Astur N Klimo P Jr Sawyer JR Kelly DM Muhlbauer MS Warner WC Jr. Traumatic atlanto-occipital dislocation in children: evaluation, treatment, and outcomes. J Bone Joint Surg Am. (2013) 95:e194. 10.2106/JBJS.L.01295 24352780

[14] Tauchi R Imagama S Ito Z Ando K Muramoto A Matsui H Surgical treatment for chronic atlantoaxial rotatory fixation in children. J Pediatr Orthop B. (2013) 22:404–8. 10.1097/BPB.0b013e3283633064 23764756

[15] Neo M Sakamoto T Fujibayashi S Nakamura T. The clinical risk of vertebral artery injury from cervical pedicle screws inserted in degenerative vertebrae. Spine. (2005) 30:2800–5. 10.1097/01.brs.0000192297.07709.5d 16371908

[16] Ling JM Tiruchelvarayan R Seow WT Ng HB. Surgical treatment of adult and pediatric C1/C2 subluxation with intraoperative computed tomography guidance. Surg Neurol Int. (2013) 4:S109–17. 10.4103/2152-7806.109454 23646272 PMC3642753

[17] Hitti FL Hudgins ED Chen HI Malhotra NR Zager EL Schuster JM. Intraoperative navigation is associated with reduced blood loss during C1–C2 posterior cervical fixation. World Neurosurg. (2017) 107:574–8. 10.1016/j.wneu.2017.08.051 28842229

